# Case report: Sustained complete remission with all-oral MEPED therapy in a patient with Hodgkin’s disease developing resistance to pembrolizumab

**DOI:** 10.3389/fphar.2024.1334233

**Published:** 2024-02-20

**Authors:** K. Reuthner, P. Aubele, K. Menhart, P. Rath, D. C. Harrer, W. Herr, J. Hahn, M. Vogelhuber, D. Heudobler, F. Lueke, A. Reichle, M. Grube

**Affiliations:** ^1^ Department of Internal Medicine III, Hematology and Oncology, University Hospital of Regensburg, Regensburg, Germany; ^2^ Medical Care Center (MVZ), Oncology, Hospital of Straubing, Straubing, Germany; ^3^ Department of Nuclear Medicine, University Hospital of Regensburg, Regensburg, Germany; ^4^ Bavarian Cancer Research Center (BZKF), University Hospital Regensburg, Regensburg, Germany; ^5^ Division of Personalized Tumor Therapy, Fraunhofer Institute for Toxicology and Experimental Medicine, Regensburg, Germany

**Keywords:** Hodgkin`s disease, relapse, pembrolizumab, resistance, MEPED, remission

## Abstract

Targeted chemotherapy and immune checkpoint inhibitors (ICPi) have expanded the spectrum of therapies for patients with relapsed/refractory (r/r) Hodgkin’s disease and significantly improved the proportion of patients with long-term disease control. However, there is no standardized therapeutic option in case of further progression. Recently, we demonstrated that therapy with MEPED (metronomic chemotherapy, everolimus, pioglitazone, etoricoxib, dexamethasone) is highly effective in patients with r/r Hodgkin’s disease. The benefit after pre-treatment with ICPi has not been studied, yet. Here, we report a patient with progressive Hodgkin’s disease on Pembrolizumab for the first time who achieved sustained complete remission (CR) after initiation of MEPED therapy. A 57-year-old patient was pre-treated with brentuximab vedotin for relapsed advanced Hodgkin’s disease and had received Pembrolizumab for progression from November 2020 to July 2022. Due to further progression, MEPED therapy was started in August 2022 and continued until May 2023. It consisted of a strictly oral daily (28-day cycle) application of low-dose treosulfan 250 mg, everolimus 15 mg, pioglitazone 45 mg, etoricoxib 60 mg, and dexamethasone 0.5 mg. Treatment response was evaluated by F-18 FDG-PET/CT (PET/CT). CR was defined by a negative Deauville score (DS) of 1-3. Already 3 months after starting MEPED, a CR (DS: 3) was confirmed by PET/CT in November 2022. The next follow-up in May 2023 continued to show CR (DS: 3). The therapy was very well tolerated. No hematological or other organ toxicity was observed. However, in May 2023 the patient presented with leg edema and weight gain, most likely due to pioglitazone and the PET/CT revealed suspected everolimus-induced pneumonitis, so MEPED was discontinued and diuretic therapy and treatment with prednisolone was started with gradual dose reduction. This resulted in a rapid complete resolution of the symptoms. The next PET-CT in July 2023 continued to show CR (DS: 3) without evidence of pneumonitis. Currently, therapy with MEPED has not been resumed. In conclusion, we demonstrate for the first time that MEPED therapy is highly effective in a patient with Hodgkin’s disease who has been refractory to ICPi. Sustained CR was achieved over 11 months after initiation of MEPED therapy. Further studies on a larger patient cohort should be performed.

## Introduction

Hodgkin’s lymphoma (HL) is a rare malignant hematological disease of the lymphatic system, which in most cases originates from B-lymphocytes (germinal-center) (Carbone et al., 2009). Characteristically, in classical HL, only a few CD30-positive malignant cells (Hodgkin-Reed-Sternberg cells, H-RS) are surrounded by a heterogeneous, non-neoplastic population of inflammatory immune cells including T- and B-cells, macrophages, plasma cells, neutrophils, eosinophils, myeloid-derived suppressor cells (MDSC) and NK cells and a variable degree of fibrosis in the tumor microenvironment (Cader et al., 2018; Connors et al., 2020; Ribatti et al., 2022).

HL occurs most commonly in adolescents and young adults, with an average age of 39 years at initial diagnosis. Depending on the subtype, a second age peak is seen in patients > 55 years (Kaseb and Babiker, 2023). Changes in the therapy of HL, such as more effective and less toxic chemotherapies, advances in radiation techniques, and improved techniques for staging (18F-fluoro-2-deoxy-D-glucose positron emission tomography, FDG-PET), have led to a marked improvement in prognosis in recent years. Risk-adapted use of combination chemotherapies and radiotherapy can now cure more than 80% of all patients even in advanced stages, making HL one of the most curable malignant diseases (Connors et al., 2020).

Depending on the stage of the disease, about 10%–30% of patients experience a relapse (Ansell, 2020). 10%–15% of patients have a refractory disease that either does not respond to primary therapy or, after initially achieving a partial remission, progresses. The goal of therapy in relapsed or refractory HL (r/r HL) is long-term remission, which can be achieved by salvage chemotherapy followed by high-dose chemotherapy with autologous stem cell transplantation (ASCT). However, with each subsequent relapse, the likelihood of achieving a sustained complete remission (CR) decreases. Patients who are not eligible for ASCT, or those who relapse after ASCT, may be treated with a rechallenge of conventional chemotherapy, targeted chemotherapy (brentuximab vedotin), immune checkpoint inhibitors (ICPi; e.g., nivolumab and pembrolizumab), or radiotherapy. Allogeneic stem cell transplantation (allo-SCT) can be curative in selected cases of r/r HL, including after prior ASCT. For patients who have already received all the currently established therapies, no further standard therapy option exists to treat disease relapse.

Recently, we demonstrated that biomodulatory MEPED therapy (metronomic chemotherapy, etoricoxib, pioglitazone, everolimus, dexamethasone) is highly effective in patients with r/r HL (Ugocsai et al., 2016; Lüke et al., 2021). In MEPED, a chemotherapeutic agent (treosulfan) is administered metronomically and in low doses, which is, therefore, better tolerated and has a continuous regulatory activity (Heudobler et al., 2019). It also includes pioglitazone which is primarily used as an oral antidiabetic agent for the treatment of type 2 diabetes mellitus. Pioglitazone is a dual peroxisome-proliferator-activated-receptor (PPAR) α/γ agonist, whose action is characterized by its agonistic effect on nuclear transcription factors and thus on the regulation of tumor growth. Furthermore, PPAR α has a pronounced anti-inflammatory effect which might be beneficial in malignant diseases (Heudobler et al., 2018). Cyclooxygenase-2 (COX2) affects the accessibility of PPAR ligands. Efficacy in the use of pioglitazone in cancer can be achieved by combining it with other master modifiers - such as COX2 inhibitors (Lüke et al., 2022). The use of other modulators of transcription factors such as glucocorticoids and the administration of metronome low-dose chemotherapy can produce a synergistic effect in tumor tissue (Heudobler et al., 2018). Furthermore, everolimus, an inhibitor of the mammalian target of rapamycin (mTOR) finds application in the MEPED regimen as the mTOR signaling pathway also plays a crucial role in malignant tissue and is often dysregulated in HL (Sun, 2021). Everolimus has recently been shown to be effective in the treatment of r/r HL (Johnston et al., 2010; Johnston et al., 2018; Mehta-Shah and Bartlett, 2018). There are already data that mTOR inhibition may support ICPi by supporting the immigration of T cells with a simultaneous reduction in the proportion of regulatory T cells (Langdon et al., 2018).

To date, the efficacy of MEPED in patients refractory to ICPi has not been studied. We report here for the first time an HL patient with multiple prior therapies who achieved sustained CR with MEPED therapy after being refractory to pembrolizumab.

## Case presentation

A 57-year-old patient was diagnosed in 2014 with stage IIISB nodular sclerosis HL according to Ann Arbor, with lymphadenopathy (left cervical, left supraclavicular, mediastinal), and splenic involvement. He reported left cervical swelling as his main complaint, B symptoms were not present. A computed tomography (CT) scan of the neck/thorax/abdomen/pelvis was performed for diagnostic imaging. The diagnosis was confirmed by histology of a cervical lymph node. The patient is known to have intellectual disability and schizophrenia due to peripartum brain damage. There was no evidence of hereditary disease and the family history was unremarkable. No other relevant medical history was known. Therapy was initiated with five cycles of chemotherapy according to the BEACOPP regimen (bleomycin, etoposide, adriamycin, cyclophosphamide, vincristine, procarbazine, and prednisolone). During cycles 4 and 5, the patient developed a urinary tract infection and pneumonia without significant clinical compromise, which were successfully treated with antibiotic therapy. A planned sixth cycle of chemotherapy could not be performed, due to repeated infectious complications as well as delayed hematopoietic recovery. CT scan showed good partial remission after 2 cycles and CR after completing therapy. Due to his disability, all chemotherapy cycles were carried out under inpatient conditions. The patient lived in a group home and worked in a sheltered workshop. The patient was in a good emotional state throughout the inpatient treatment. Over 6 years of regular follow-up, which included an unremarkable medical history, physical examination, and laboratory tests (including blood counts, kidney and liver function tests, and inflammatory parameters), there was no evidence of disease recurrence.

In February 2020, the patient complained of left cervical swelling. FDG-PET combined with computed tomography (PET/CT) showed lymphadenopathy bilaterally cervical and periclavicular and in the left mediastinum. The laboratory tests revealed no abnormalities. In addition to the suspected recurrence of HL, lymphadenopathy of other causes (e.g., infectious, autoimmune, or other malignancy) was also considered in the differential diagnosis. However, a histologic examination of a left cervical lymph node revealed a relapse of the previously known HL. Since the patient, due to intellectual disability and schizophrenia was not suitable for ASCT, targeted chemotherapy with brentuximab vedotin was initiated. The therapy was well tolerated, and no dose modification was required. In October 2020, after seven cycles of therapy, the patient showed progression. As the progression was at a previously histologically confirmed site, a new biopsy was not performed this time. Therefore, in November 2020, the therapy was switched to pembrolizumab. Hereby, partial remission was achieved. This therapy was also well tolerated. In April 2022, after 24 applications, PET/CT again showed disease progression (left cervical lymphadenopathy, Deauville score (DS): 4). At that time, the patient had no complaints. Due to intellectual disability, he was not eligible for allogeneic stem cell transplantation. Due to the advanced stage of the disease and the fact that the patient’s treatment options were limited, we were looking for a therapy that would allow HL to respond and maintain quality of life for as long as possible.

After approval by the health insurance and consent from the caregiver, therapy with MEPED was started in August 2022.

MEPED therapy consisted of a strictly oral and daily application of treosulfan 250 mg, everolimus 15 mg (with a target serum level of 15 ng/mL), pioglitazone 45mg, etoricoxib 60mg, and dexamethasone 0.5 mg (Table 1). The medication was taken from day 1-28 (28-day cycle). All the drugs were approved by the European Medicines Agency (EMA). To assess treatment response, PET/CT was performed before and during ongoing therapy. CR was defined by a DS of 1-3. As with the previous therapies, the PET/CT scan was a challenge for the patient because it had to be done under anesthesia due to his intellectual disability. During MEPED therapy, clinician-assessed outcomes were collected at regular appointments by performing clinical examinations, taking the patient’s medical history, and determining vital signs and laboratory tests (including blood counts, kidney and liver function tests, and inflammatory parameters). Due to the patient’s intellectual disability, the collection of patient-assessed outcomes was limited and no specific tools could be used.

**TABLE 1:** MEPED-Regimen (28-day cycle).

Drug name	Dose (mg)	Days applicated	Comments
Pioglitazone	45	1–28	
Treosulfan	250	1–28	Mild antiemetic on demand (i.e., metoclopramide)
Everolimus	15	1–28	To achieve nadir levels of 15 ng/mL
Etoricoxib	60	1–28	
Dexamethasone	0.5	1–28	

Drugs used as part of MEPED therapy, their dosage, and duration of application.

**FIGURE 1:**
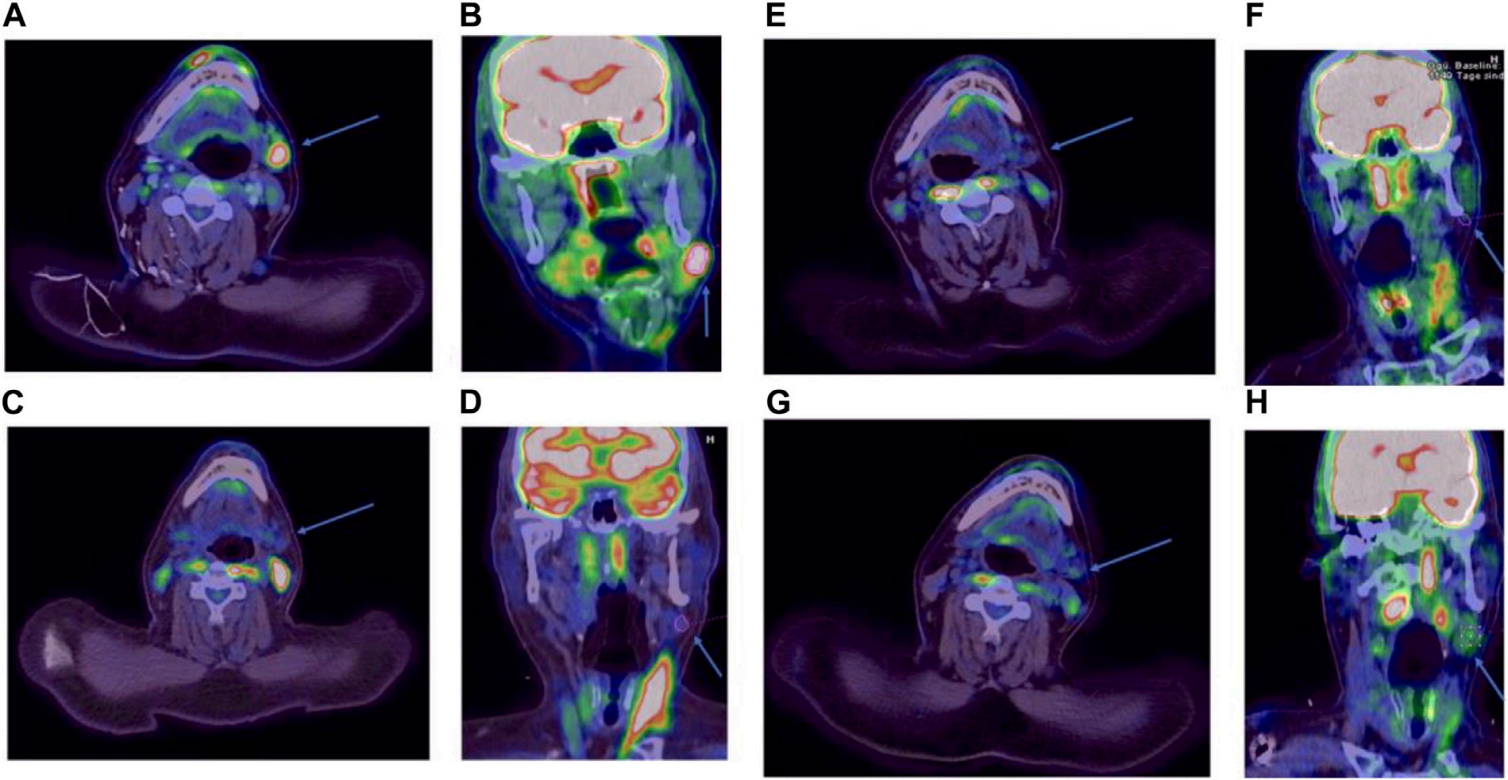
PET/CT images in axial and coronal reconstruction. The target lesion is indicated by an arrow. FDG avidity is expressed by the Deauville score (DS). Before initiation of MEPED, DS: 4 **(A, B)**. After 3 months of therapy, DS: 3 **(C, D)**. After 9 months of therapy, DS: 3 **(E, F)**. Two months after discontinuation of therapy, DS: 3 **(G, H)**.

**FIGURE 2:**
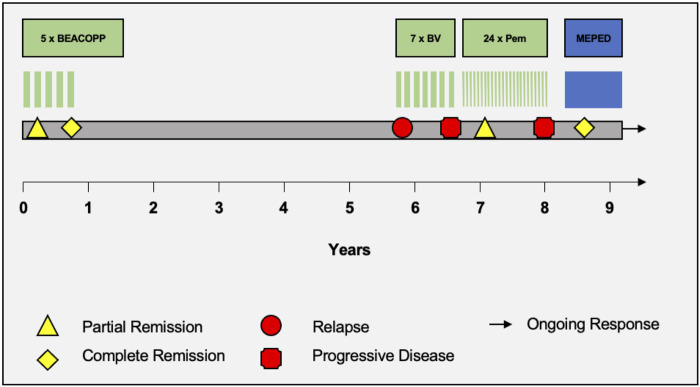
Therapy timeline. Schematic portrayal of the different therapy regimens in chronological order. BEACOPP = bleomycin, etoposide, doxorubicin, cyclophosphamide, vincristine, procarbazine, prednisone. BV = brentuximab vedotin. Pem = pembrolizumab. MEPED = treosulfan, everolimus, pioglitazone, etoricoxib, dexamethasone.

Already 3 months after starting MEPED, a CR could be confirmed by PET/CT imaging (DS: 3) in November 2022. The therapy was well tolerated. The patient was not affected in his daily life and was able to live in his group home and work in the sheltered workshop. No dose adjustment was required in the absence of hematologic or other organ toxicities. At the next follow-up in May 2023, the patient continued to show sustained CR (DS: 3). However, the patient presented in a slightly reduced general condition (Karnofsky index of 80%) with leg edema and significant weight gain and reported mild exertional dyspnea. Echocardiography showed a normal left ventricular ejection fraction (LVEF) and no evidence of diastolic dysfunction or structural changes. There were no abnormalities on the ECG. Since there were no signs of acute heart failure in the examinations, pioglitazone was considered the most likely cause of the edema and was therefore discontinued. Recompensation was rapidly achieved by diuretic therapy. PET/CT also showed bilateral pulmonary infiltrations. However, inflammatory parameters (CRP) were normal and the patient had no symptoms of infection. In the absence of laboratory and clinical evidence of infection, bronchoscopy was not performed for further evaluation. Drug-toxic pneumonitis was therefore suspected, with everolimus as the most likely causative agent. MEPED was therefore completely discontinued and empirical therapy with prednisolone (absolute 20 mg with gradual dose reduction) was initiated. This resulted in a rapid complete resolution of the symptoms and the patient was able to return to his normal daily life. The next PET/CT in July 2023 continued to show CR (DS: 3) and no evidence of pneumonitis (PET/CT images illustrating the response are shown in Figure 1). The patient was in good general condition and without any complaints. Currently, MEPED has not been resumed. The different therapy regimens in chronological order are shown in Figure 2.

## Discussion

In recent years, therapeutic options for r/r HL have been significantly expanded by targeted chemotherapy (brentuximab vedotin) and ICPi (pembrolizumab, nivolumab) (Mehta-Shah and Bartlett, 2018).

However, not all patients are suitable for these therapies (e.g., severe autoimmune disease or other comorbidities) and there are no established therapies after failure of these therapeutic approaches.

Recently, we demonstrated that biomodulatory MEPED therapy can achieve a very significant response to sustained CR in patients who were refractory to both intensified salvage or targeted chemotherapy with brentuximab vedotin and high-dose chemotherapy with ASCT (Ugocsai et al., 2016; Lüke et al., 2021). Some patients have even been able to undergo curative allogeneic stem cell transplantation with this bridging therapy and are in sustained CR. Furthermore, biomodulatory therapy has also been shown to successfully induce remission in early relapsed acute myeloid leukemia (AML) after allogeneic stem cell transplantation (Kattner et al., 2020).

The use of MEPED therapy in HL patients who are refractory to therapy with ICPs has not yet been investigated. In the present case, we report for the first time a patient with r/r HL who progressed on therapy with pembrolizumab and rapidly achieved sustained CR with MEPED therapy. Due to a limited general condition and preexisting brain injury with schizophrenia, the patient was not suitable for intensive salvage therapy like high-dose chemotherapy with ASCT or allo-SCT. Therefore, it was important to establish a therapy that had a favorable safety and toxicity profile and did not impose too much burden on the patient’s daily life. MEPED therapy was well suited for this patient because it was a strictly oral therapy that could be administered entirely on an outpatient basis and did not burden the patient with infusions or frequent hospitalizations. The patient tolerated the therapy very well for the first 9 months, with no hematological or other organ toxicities, and no dose adjustments were required. During the entire therapy, he was not affected in his daily life and was able to live in his group home and continue his job in the sheltered workshop. However, after 9 months of therapy, the patient experienced significant weight gain and developed leg edema most likely due to pioglitazone, but this was rapidly resolved with diuretic therapy. At that time, the patient also reported mild exertional dyspnea and the PET/CT showed signs of pneumonitis, which was most likely interpreted as toxic pneumonitis related to everolimus. Therefore, MEPED therapy was discontinued. However, temporary and tapering therapy with prednisolone resulted in a swift complete regression of the pulmonary changes. As a result, the patient was completely symptom-free and able to return to his normal daily life.

In line with previous reports, MEPED therapy is therefore a well-tolerated regimen with a good safety profile. This is particularly important for HL patients, as the onset of the disease is usually in younger adulthood, and toxicities and second malignancies can be a challenge, particularly later in life when new therapies are required (van Leeuwen and Ng, 2016). Especially in r/r HL patients with impaired general condition or elderly patients with comorbidities who are not eligible for intensive salvage or toxic consolidation therapy, MEPED is a well-suited treatment modality to achieve sustained remission (Evens et al., 2019; Carter et al., 2020). However, patients should be closely monitored for potential side effects (e.g., weight gain and edema due to pioglitazone or everolimus-induced pneumonitis) so that therapy can be interrupted or discontinued if necessary.

In addition to the favorable safety profile and the advantage of all-oral application, MEPED represents a cost-effective therapy compared to many other treatments (approx. 3,000 Euro per month).

The therapeutic effect of MEPED in our patient not only occurred very quickly but could also be maintained over a long period of time. Already 3 months after the start of MEPED, a CR could be detected in the PET/CT, which in the meantime could be maintained over 11 months. The fact that CR could also be detected after discontinuation of MEPED suggests that the biomodulatory effect is longer-lasting. So far, it is unclear how long MEPED should be used to maintain remission, however, long-term follow-up studies have shown remission rates of 83% in patients with r/r HL (Lüke et al., 2021).

With pembrolizumab, the patient achieved at most a partial remission, whereas with MEPED, a CR was achieved after only 3 months. Recently, components of MEPED have been described to have a positive effect on T-cell function and may induce upregulation of immune checkpoints (Hirayama et al., 2016; Chowdhury et al., 2018; Renner et al., 2019; Batyrova et al., 2020; Lüke et al., 2021). It could be speculated that the use of MEPED may be particularly beneficial in the post-ICPi sequence, as it could lead to a reversal of the resistance mechanisms induced by ICPi and a restoration of T-cell function in tumor tissue. In several tumor types, chemotherapy following ICPi therapy has been shown to improve tumor response and there is evidence that ICPi therapy may increase sensitivity to subsequent chemotherapy (Schvartsman et al., 2017; Park et al., 2018; Szabados et al., 2018; Hadash-Bengad et al., 2020). In addition to their cytotoxic effects, chemotherapeutic agents, even when administered metronomically as in MEPED therapy, have been shown to have immunostimulatory effects by targeting tumor cells or cells of the innate and adaptive immune system (e.g., macrophages, NK cells, dendritic cells, and T-cells) which may result in synergistic and additive effects to ICPi pre-treatment (Bracci et al., 2014; Galluzzi et al., 2020; Muraro et al., 2023).

## Conclusion

In summary, biomodulation with MEPED is a beneficial and cost-effective treatment option with the potential for rapid induction of durable remission even in patients with r/r HL who are refractory to pretreatment with ICPi. The oral route of administration has the advantage that patients do not require regular inpatient or outpatient treatment. Its favorable safety and tolerability profile also makes it suitable for patients with poor general health or co-morbidities. To better understand the impact of MEPED therapy on r/r HL patients in ICPi-refractory scenarios, larger prospective studies are necessary. In addition, the pathophysiological mechanisms of the therapy should be further analyzed.

## Data Availability

The original contributions presented in the study are included in the article/Supplementary material, further inquiries can be directed to the corresponding authors.
